# A Theoretical Model for Structural Health Monitoring of the Thermal-Mechanical Behaviour of Plates Under Combined Laser Irradiation and Airflow

**DOI:** 10.3390/ma19204268

**Published:** 2026-10-09

**Authors:** Qi Pu, Xiaodong Jia, Yizhuo Gui, Shaozun Hong, Te Ma, Hongwei Song, Cheng Qiu

**Affiliations:** 1 High Speed Aerodynamics Institute, China Aerodynamics Research and Development Center, Mianyang 621000, China; 2 Key Laboratory for Mechanics in Fluid Solid Coupling System, Institute of Mechanics, Chinese Academy of Sciences, Beijing 100190, China

**Keywords:** thermal stress, mechanical analysis, theoretical model, machine learning, explainable AI

## Abstract

Laser irradiation, as a high-energy input, induces a rapid temperature rise on a structure’s surface, resulting in a localized stress concentration, which is one of the key elements in catastrophic failure. Accurate structural health monitoring of plates should take stress distribution into account. In this paper, we present a theoretical model for determining the stress state of a plate under combined laser radiation and airflow, using temperature values from several discrete measurement points as input. First, a fitting function was proposed based on data-driven thermal analysis, which is utilized to acquire the temperature distribution on the whole surface. The stress state near the area of the laser radiation was then calculated using a theoretical model based on the elasticity theory of an axis-symmetric problem. With the incorporation of the data-driven method, the model is then extended from a baseline analytical model to account for three-dimensional heat-conduction effects and the nonlinear temperature dependence of thermophysical properties. The effectiveness and precision of the proposed model were verified by coupled thermal-mechanical simulations. The current work can provide substantial support for developing a structural health monitoring system for aerospace structures exposed to laser radiation.

## 1. Introduction

High-energy laser technology has become a crucial part of advanced manufacturing, aeronautical engineering, and defense applications [1,2,3]. The interaction between laser irradiation and materials is a major physical concept underlying many applications, and can cause significant thermo-mechanical damage and failures. Powerful laser heating can produce non-uniform temperature fields, which, when combined with external mechanical loads and environmental effects, can cause significant deformation, buckling, and even breakage of load-bearing structures [4,5].

A growing amount of experimental and numerical evidence shows that laser-induced thermal stress and deformation play a significant role in structural failure, particularly in aerodynamic conditions. Boley et al. [6] explored laser irradiation of metallic objects subjected to tangential airflow and discovered that thin metallic plates could break before reaching melting temperatures. Wang et al. [7] investigated the “avalanche” phenomenon in laser ablation of C/SiC composites under hypersonic airflow and showed that the ablation rate was significantly higher than under static-air conditions.

However, the instant and ultra-high-temperature nature of laser–material interaction makes real-time measurement of thermo-mechanical fields under laser irradiation difficult. Yuan et al. [8] used high-temperature speckle pattern preparation and optical filtering to measure the dynamic deformation process and high-temperature thermo-buckling morphology of aluminum plates at low laser powers. Sakai et al. [9] developed an in situ ablation sensor for real-time measurements at ablation interfaces, but its measurement area was limited. Tang et al. [10] used a high-power blue light source and a blue bandpass filter to reduce high-temperature radiation interference, allowing them to observe the surface evolution of C/C composites in real time during ablation below 2000 °C. Ma et al. [11] recently established an in situ observation approach for collecting laser-induced extreme-temperature ablation morphologies and explored the dynamic ablation behavior of metallic and composite materials under supersonic tangential airflow using image processing techniques. While these studies have primarily focused on overcoming optical filtering issues for high-temperature deformation/ablation observation, high-accuracy strain measurements under laser irradiation have yet to be fully addressed.

Obtaining reliable full-field temperature measurements during laser exposure remains challenging. Infrared thermography is an important non-contact approach, but the inferred temperature depends on surface emissivity, reflected radiation, spectral response, and radiometric calibration [12]. Specialized optical methods, including colorimetry, have also been used for laser-irradiated structures [8]. These measurement requirements motivate the present reconstruction of the temperature field and associated mechanical response from discrete temperature observations.

To address the scarcity and difficulty of direct measurements, inversion strategies that infer key quantities (e.g., laser parameters and thermo-mechanical response) from limited observations have received increasing attention. Magalhães et al. [13] developed a nonlinear three-dimensional heat conduction model for fiber-laser welding and used time-traveling regularization to extract heat-flux distribution from thermocouple data. Data-driven methods have been applied to materials research [14] and to predicting the surface response of composites in extreme environments [15]. Such approaches are also used to extract structural health information from measurements [16]. Raissi et al. [17] introduced physics-informed neural networks that incorporate governing partial differential equations into data-driven solutions of forward and inverse problems. Maji et al. [18] established an integrated neural network and adaptive neuro-fuzzy inference model to predict deformation responses using laser power and spot size as inputs. Billah et al. [19] investigated an inverse transient heat-transfer problem for aluminum plates, employing neural networks to reconstruct unknown heat flux on one surface from measured temperature data on another. Lu et al. [20] used numerical heat-transfer solvers and deep learning to convert discrete temperature observations to boundary conditions. Du et al. [21] investigated multi-parameter inversion for oblique-incidence laser interaction using multivariate thermo-mechanical responses. Improvements in structural health monitoring (SHM) indicate that reconstructing thermomechanical state variables from sparse thermal sensor networks can improve health evaluation when combined with proper material or structural failure criteria. Motivated by this need, the goal of this research is to develop a temperature-based thermoelastic response reconstruction model for plates subjected to laser irradiation and airflow. Discrete temperature observations are used to reconstruct the temperature and in-plane strain fields. Interpretable machine learning further identifies explicit relations between the fitted temperature-field parameters and the laser parameters. These relations provide a basis for recovering laser parameters from temperature observations under the modeled conditions.

This paper is organized as follows. Section 2 describes the thermal model and temperature-field fitting. Section 3 presents the analytical mechanical model, numerical comparisons, a quantitative thickness correction derived by interpretable machine learning, and preliminary examples with temperature-dependent material properties. Section 4 summarizes the conclusions.

## 2. Thermal Analysis of Temperature Fields

### 2.1. Numerical Model Description

Since the aim of the paper is to establish a model for analyzing thermal and mechanical behavior from discrete measuring points on the back surface, the first step here is to infer a temperature field from limited data. In order to do so, a data-driven approach is proposed in this section. The basic idea of the data-driven approach is that a quantitative model is built to bridge the connection between data from the sensors and the whole temperature field, which is a typical regression task in the field of machine learning. With such a regression model, we then use techniques of explainable artificial intelligence (XAI) to form a mathematical relation to quickly capture the temperature field.

**Figure 1:**
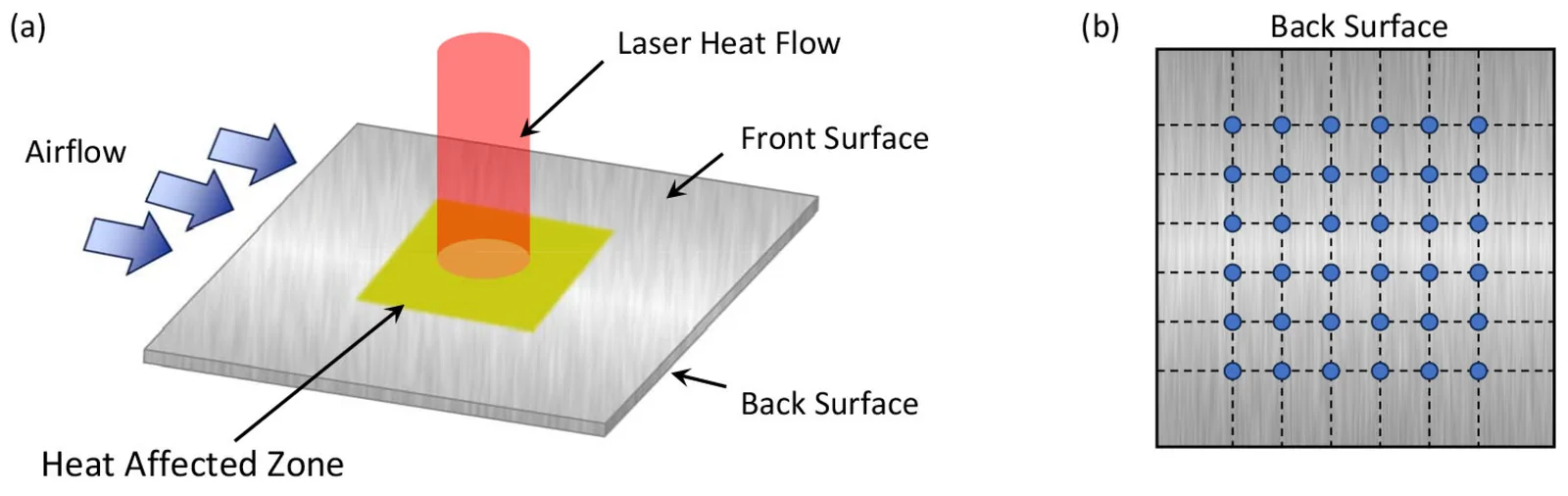
(**a**) Schematics of the thermal analysis of the plate under combined laser radiation and airflow. (**b**) Locations of the temperature measuring points.

As a data-driven approach, the above campaign was performed on the basis of a numerical dataset. The numerical model is illustrated in Figure 1a. The size of the plate is 100 mm × 100 mm, with a thickness of 5 mm. The loading conditions, as shown in the figure, contain a laser heat flow and an airflow, both of which are applied to the front surface of the plate. The 6×6 measuring points are assumed to be distributed on the back surface of the plate with a space interval of 12.5 mm, as a typical practice in experiments. The position and the radius of the laser spot are set to be random variables to account for the unknown uncertainties, with the central point of the laser randomly located in a range within 10 mm of the plate center, the diameter in the range of 10 mm to 50 mm, and the laser power ranging from 2 kW to 4 kW. All the temperature data within an irradiation time of 3 s are recorded.

The material properties included in the heat transfer analysis are heat conductivity of 160 W/(m· K) and specific heat of 900 J/(kg· K).

The configuration in Figure 1 represents an idealized condition relevant to studying the protection of high-speed vehicle structures against potential high-energy laser exposure. The material is subjected to a combined thermal boundary condition consisting of localized laser heat flux and airflow-related convective heat exchange. The airflow represents the external environment over the structural surface. Its modeled effect is forced convection. Aerodynamic pressure and shear-traction loads are not included in the analytical mechanical formulation.

Instead of running a direct CFD simulation, a simplified heat-transfer model was utilized in Abaqus to reduce the computational cost of large-scale data generation. The modeling procedure is summarized as follows:

- A first-step heat transfer analysis
  A transient heat-transfer analysis was performed to solve heat conduction within a three-dimensional aluminum plate. The laser source was represented by a top-hat profile, with a uniform absorbed heat flux inside the circular spot and zero laser heat flux outside it. During the irradiation interval, the imposed heat flux is
  $$q_{L}\left(r_{L}\right)=\left\{\begin{array}{cc} \frac{\eta P}{A_{\mathrm{spot}}}=\frac{4\eta P}{\pi d^{2}}, & 0\leq r_{L}\leq d/2,\\ 0, & r_{L}>d/2. \end{array}\right.$$
  Here, $r_{L}$ is the distance from the laser-spot center, *d* is the spot diameter, $A_{\mathrm{spot}}=\pi d^{2}/4$ is the spot area, and *P* is the incident laser power. The plate absorptivity was set to the constant value η=0.3 in all simulations. Thus, the absorbed laser power is 0.3P. The top-hat assumption applies to the imposed laser heat flux. The Gaussian function introduced in Section 2.2 describes the resulting temperature field after heat conduction and convective exchange.
  Forced convection induced by an external airflow was represented using Film Condition boundary conditions, with a convection coefficient *h* estimated from classical flat-plate correlations. In this baseline configuration, the free-stream (sink) temperature was initially set to the ambient value $T_{\infty }$.
- Spatial segmentation of the convective boundary
  The constant *h* and $T_{\infty }$ cannot account for the airflow heating and the resulting downstream variation of the cooling potential. In this regard, the top surface was partitioned into multiple sub-regions, with $N_{x}$ segments along the streamwise direction and $N_{y}$ segments along the transverse direction. This partitioning enables the assignment of region-dependent sink temperatures in subsequent iterations.
- Iterative update of the effective sink temperature
  An iterative scheme was adopted to update the sink temperature in each surface sub-region based on the wall temperature obtained from the previous Abaqus solution, thereby emulating the progressive heating of the near-wall airflow. The updated airflow temperature is estimated based on energy balance:
  (1) $$T_{air}^{\left(i+1\right)}=T_{air}^{\left(i\right)}+\frac{hA(T_{w}^{\left(i\right)}-T_{air}^{\left(i\right)})}{\rho U\delta \Delta yc_{p}}$$
  (2) $$\delta =0.37x/Re_{x}^{0.2}$$
  $T_{w}^{\left(i\right)}$ and $T_{air}^{\left(i\right)}$ are the wall and air temperatures in the sub-region (with the area of *A*) from the previous iteration. ρ and *U* are the density and velocity of the airflow. δ is the estimation of the thickness related to mass flow rate and Δy is the length of the sub-region along the transverse direction. $c_{p}$ is the specific heat.
- Dataset generation and outputs
  The final converged (or fixed-iteration) temperature fields were exported as the numerical dataset for subsequent data-driven modeling. The outputs include the full-field temperature distribution as well as temperatures at predefined sampling grids on the back surface.

The coupling represented by this procedure is limited to the prescribed thermal-loading description. The wall temperature updates the effective air temperature through Equation (1), and the updated air temperature enters the subsequent heat-transfer solution. This is a simplified form of thermal feedback within the convective boundary condition. The airflow velocity and laser heat input remain prescribed, and changes in the flow field or laser deposition caused by deformation, ablation, or other surface evolution are not solved. Temperature drives the mechanical response, without deformation being fed back into the heat-transfer calculation. These omitted mechanisms are not assumed negligible for all aerospace laser-exposure conditions. Situations in which they materially change the thermal loading or material state are outside the scope of the present model.

The three-dimensional thermal model is not axisymmetric. The directed airflow and the spatially updated convective boundary condition introduce a streamwise asymmetry, even for a circular laser spot. The axisymmetric approximation is introduced only in the subsequent mechanical reconstruction, based on the nearly radial temperature distribution in the localized heated region.

**Figure 2:**
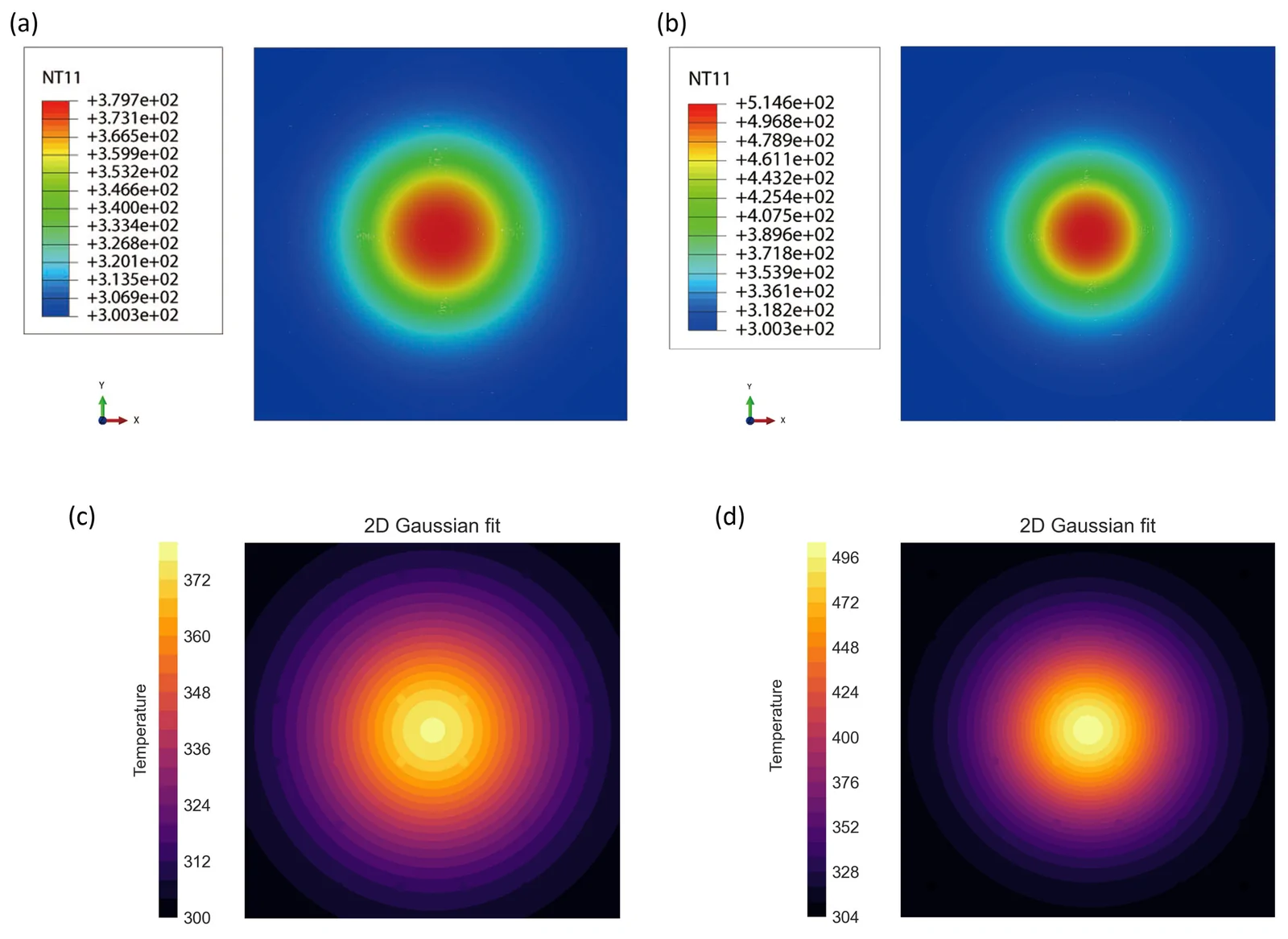
Typical temperature fields on the back. Case 1: P = 2000 W, d = 40 mm, Ma = 0.6, t = 3 s. Case 2: P = 4000 W, d = 30 mm, Ma = 1, t = 3 s. (**a**) Case 1: simulation. (**b**) Case 2: simulation. (**c**) Case 1: fitted results. (**d**) Case 2: fitted results.

The modeling procedures were performed via a Python 3.8 script run in Abaqus 2021. The script automatically runs cases with different parameters, including airflow speed and laser diameter. Then, the temperature data of each case was saved after the job was finished. The typical full-field temperature and temperatures at the sampling grid under combined laser irradiation and aiflow are shown in Figure 2.

The simulation cases presented in the validation figures were not used to train the corresponding machine learning models. They therefore constitute unseen cases for evaluating the learned relations and the response reconstruction. The training samples illustrated in Figure 2 are distinct from these validation cases. Fitting the Gaussian temperature field from the discrete temperature samples of a new case is part of applying the reconstruction procedure. These comparisons provide numerical validation on unseen cases.

### 2.2. Fitting Function by Data-Driven Analysis

Multiple examples such as that in Figure 2 indicate that the temperature field is very near to a Gaussian function, despite the fact that the energy distribution characteristics of the laser point do not match this shape. As a result, the following equation will be used to fit the temperature field.

(3) $$f\left(x,y\right)=A\cdot \exp\left[-\frac{{\left(x-\mu_{1}\right)}^{2}}{2\sigma_{x}^{2}}-\frac{{\left(y-\mu_{2}\right)}^{2}}{2\sigma_{y}^{2}}\right]+B$$

**Figure 3:**
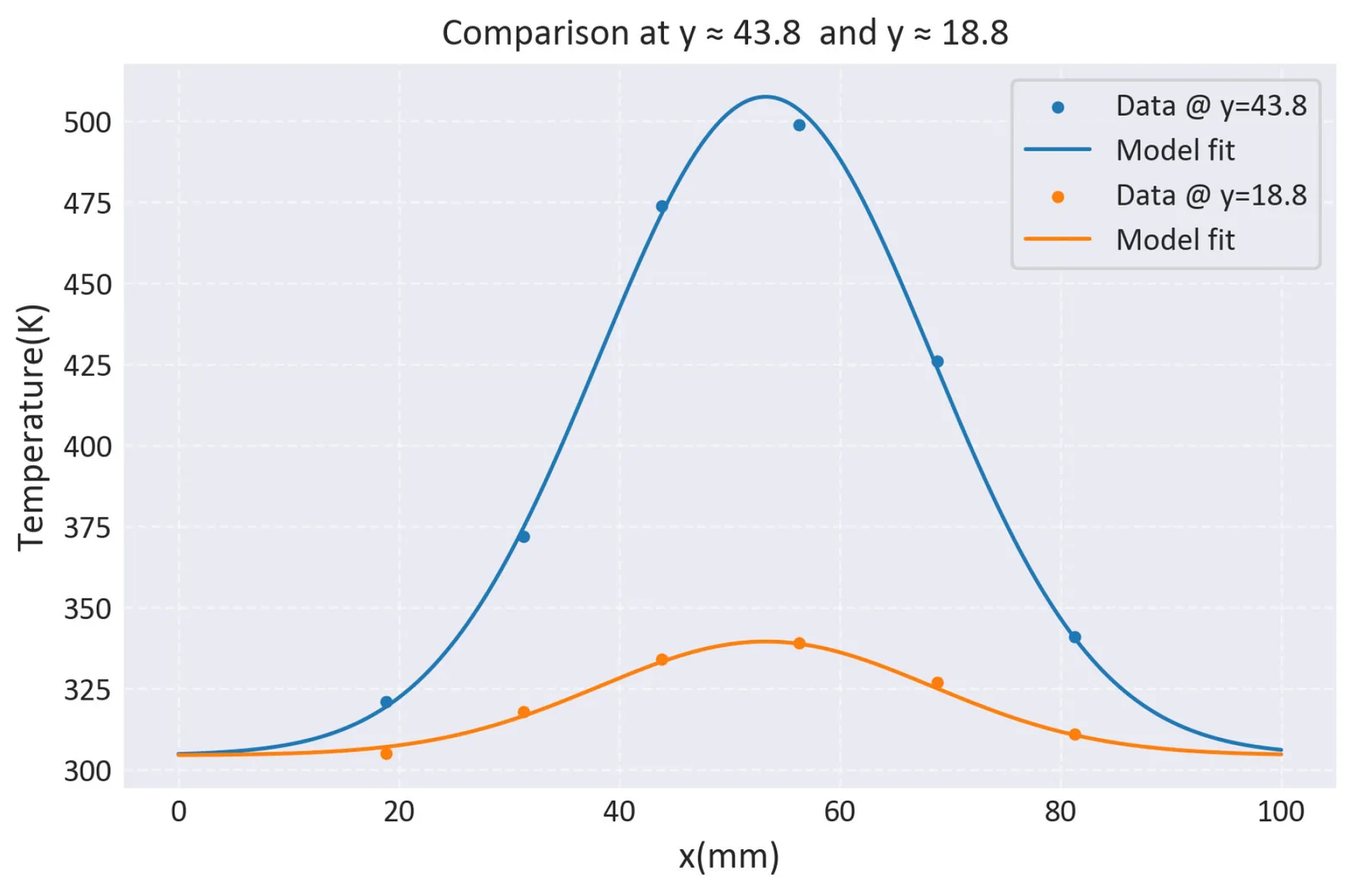
Comparison between the fitting function and the original data.

The fitting was done in Python with SciPy’s non-linear least-squares technique, which minimized the sum of squared residuals between the model and the measured data. The fitted center and widths describe the location and spatial extent of the reconstructed temperature distribution. Their quantitative relations with the laser parameters, established below by symbolic regression, allow this temperature information to be used for laser-parameter estimation. The comparison between the fitting function and the original data is presented in Figure 3. Data points at the bottom and the central part of 6×6 measuring points are illustrated. Both of the R-square values of the regression performance are over 0.99, showing that Equation (3) is an adequate equation for determining the temperature field.

**Figure 4:**
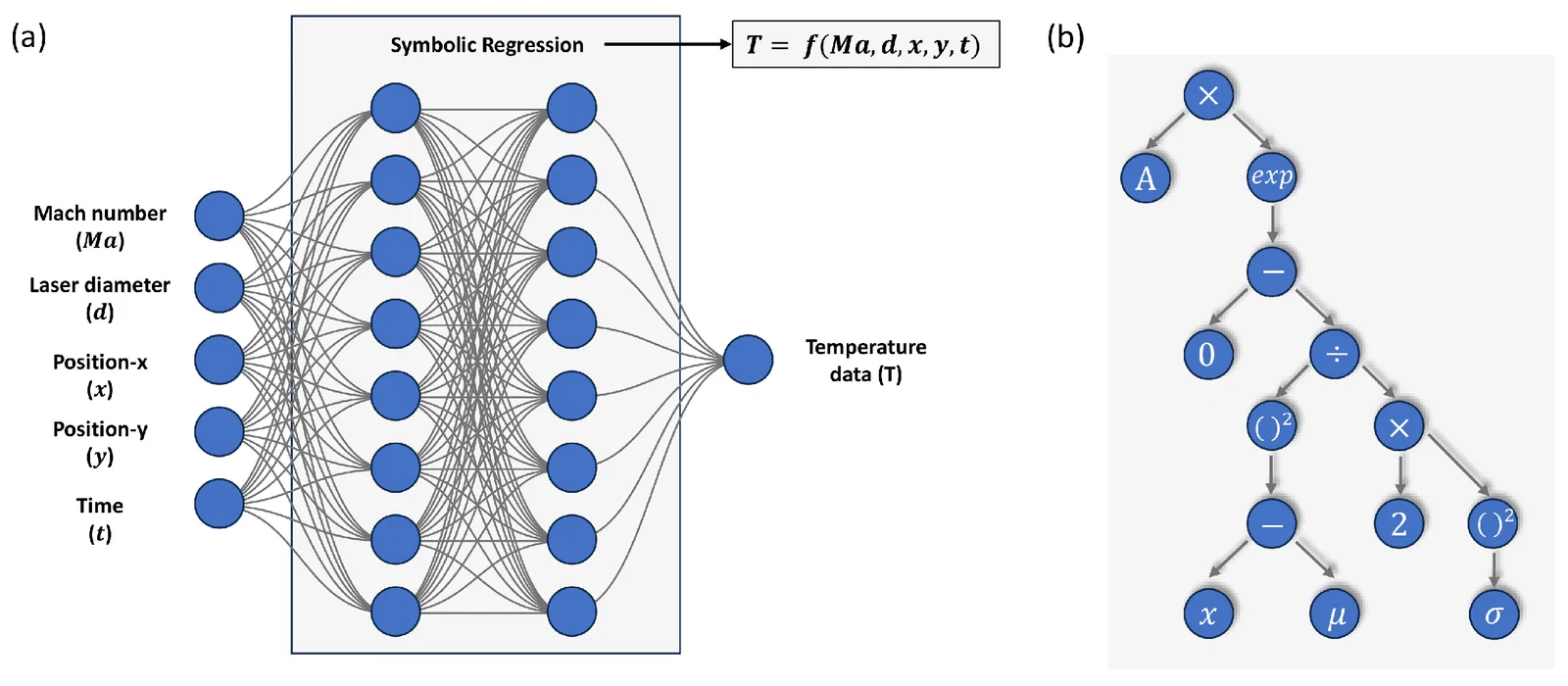
(**a**) The data-driven analysis for the temperature field using the symbolic-regression model. (**b**) Hierarchical expression tree representation of the Gaussian function $f\left(x\right)=A\cdot \exp\left(-\frac{{\left(x-\mu \right)}^{2}}{2\sigma^{2}}\right)$ in the symbolic-regression model.

The numerical training data are used to identify explicit quantitative relations between the laser conditions and the parameters of the fitted temperature field. These relations describe the thermal response and can also be rearranged for laser-parameter recovery. The model for analyzing the temperature field is trained as illustrated in Figure 4. The model is essentially a symbolic-regression (SR) model, which is an interpretable machine learning technique that discovers analytical expressions directly from data through evolutionary algorithms. Similar to the genetic optimization process, the method initializes a population of candidate formulas represented as hierarchical expression trees (as presented in Figure 4b) combining mathematical operators (+, −, ×, ÷, exp, etc.), variables, and constants in prefix notation. Through successive generations, fitter individuals are gradually selected for genetic operations, which refer to the crossover, mutation, and reproduction among the parts of the trees. A Pareto front was constructed to characterize the trade-off between equation complexity and prediction error, and a representative optimal expression was selected from this front as the final model. In our study, the SR model was built and implemented via PySR, an open-source code library, to identify the governing equations for the temperature distribution on the plate under laser irradiation.

**Figure 5:**
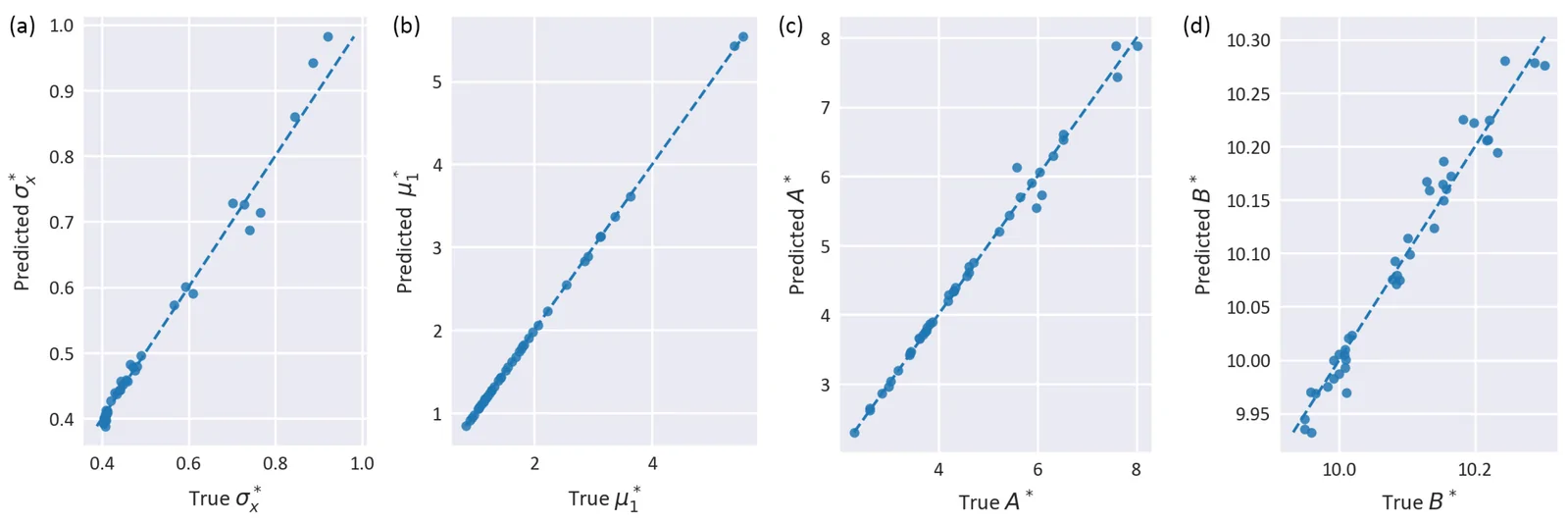
Regression performance of the equations by the SR model for determining the temperature field. The normalized values of (**a**) $\sigma_{x}^{*}$, (**b**) $\mu_{1}^{*}$, (**c**) $A^{*}$, and (**d**) $B^{*}$.

The fitted Gaussian parameters were analyzed using symbolic regression. A PySR-based symbolic-regression model was trained to search for compact closed-form expressions that map the dimensionless inputs to the dimensionless output. Non-dimensionalizing both inputs and outputs enforces dimensional consistency and isolates the governing scaling behavior, thereby improving the interpretability and generalizability of the symbolic-regression model. Specifically, as an example, the Gaussian width parameter $\sigma_{x}$ was non-dimensionalized as $\sigma_{x}^{*}=\sigma_{x}/d$, while the dimensionless inputs include the normalized laser power p∗ ($p^{*}=p/p_{0}$, $p_{0}=3$ kW), spot diameter $d^{*}$ ($d^{*}=d/d_{0}$, $d_{0}=30$ mm), and Mach number. Equation (4) was obtained by the SR model, with a good $R^{2}$ score of 0.98 in the test dataset indicating the strong quantitative relationship between $\sigma_{x}^{*}$, the shape parameter of the Gaussian fitting, and the spot diameter $d^{*}$. This can be reasonably expected, as the heat exchange caused by the airflow is much smaller than the heat flux density of the laser, making the Mach number negligible in the overall shape of the temperature distribution. The regression performance of Equation (4) is shown in Figure 5a. Similar procedures can be utilized to find the equations of $\sigma_{y}^{*}$, $\mu_{1}^{*}$, $\mu_{2}^{*}$, $A^{*}$, and $B^{*}$, which relate to the position of the laser spot ($x_{laser}$, $y_{laser}$), the power, the diameter of the laser spot, and the Mach number in different ways, as shown in Equations (5)–(8). Their regression performance is shown in Figure 5b–d, which suggest a good agreement between the predictions and the true values.

(4) $$\sigma_{x}^{*}=\sigma_{y}^{*}=0.273{\left(1+\frac{0.296}{d^{*}}\right)}^{2}$$

(5) $$\mu_{1}^{*}=x_{laser}^{*}$$

(6) $$\mu_{2}^{*}=y_{laser}^{*}$$

(7) $$A^{*}=p^{*}\cdot \left(7.528{\left(1-0.365d^{*}\right)}^{2}+1.94\sqrt{1-0.266Ma}\right)$$

(8) $$B^{*}=-0.152p^{*}\left(-p^{*}+{\left(d^{*}\right)}^{2}\right)+10.1$$

Some key physical trends can be found from these equations. The $A^{*}$, which stands for the local temperature rise, scales approximately linearly with the normalized laser power $p^{*}$ and decreases with the increasing spot diameters $d^{*}$, implying that the peak temperature rise is primarily energy-input controlled. The $\sqrt{1-0.266Ma}$ term further suggests Ma acting as secondary modifiers through convective heat removal. The $B^{*}$, which stands for the average temperature rise, implies a non-monotonic dependence on power and spot size. For a fixed $d^{*}$, the quadratic form yields a minimum at $p^{*}/d^{*2}=0.5$, which can be equivalently expressed as a critical normalized intensity. This suggests that the baseline temperature level is governed by a competition between power-driven heating and spot-size-induced spatial spreading, leading to an optimal (minimum) offset near this critical combination rather than a strictly monotonic trend.

Equations (4)–(8) provide explicit relations that can be used for laser-parameter recovery from a fitted temperature field within the sampled parameter range. In particular, Equation (4), together with $\sigma_{x}^{*}=\sigma_{x}/d$ and $d^{*}=d/d_{0}$, gives

$$d^{*}=\frac{H+\sqrt{H^{2}-4{\left(0.296\right)}^{2}}}{2},\;H=\frac{\sigma_{x}}{0.273d_{0}}-2\left(0.296\right).$$

The larger root shown here corresponds to the stated spot-diameter range of 10–50 mm, for which $d^{*}>0.296$ with $d_{0}=30$ mm. The laser position follows from Equations (5) and (6). For a known Mach number, Equation (7) can then be rearranged to recover the normalized laser power as

$$p^{*}=\frac{A^{*}}{7.528{\left(1-0.365d^{*}\right)}^{2}+1.94\sqrt{1-0.266Ma}},\;P=p_{0}p^{*}.$$

The fitted parameters must use the same normalization as the symbolic-regression model. The recovered values must satisfy the stated parameter ranges and the real-valued domains of the expressions. Equation (8) supplies an additional relation for checking consistency with the fitted temperature offset. These algebraic relations make laser-parameter recovery part of the framework. The regression comparisons assess the accuracy of the learned parameter relations. They do not by themselves quantify the sensitivity of the recovered laser parameters to measurement noise.

The symbolic-regression relations in Equations (4)–(8) describe the fixed irradiation time t=3 s. Time and dimensionless time were not included as regression inputs. At another time, the same reconstruction procedure can be applied by fitting Equation (3) to the temperature observations at that instant and then evaluating the strain field under the same quasi-static mechanical assumptions. Its applicability depends on whether the evolving temperature field remains well represented by Equation (3) and sufficiently close to radial symmetry for the mechanical approximation. The laser parameter relations and the learned thickness correction must be recalibrated or independently assessed before they are used at other times.

## 3. Theoretical Model of Mechanical Behavior

### 3.1. Model Description

The rise in temperature on the structural surface would lead to stress concentration, which may cause failure prior to the complete ablation of the material, as it can be weakened under high temperatures. Meanwhile, high temperature would result in the potential malfunction of strain sensors, which makes it hard to measure the strain directly. In this context, it is of great significance to find out the stress or strain caused by the heat effect of laser radiation. In this section, we propose a theoretical model for calculating the strain distribution based on the known temperature field.

The loading case follows Figure 1a: a plate of dimensions 100 mm × 100 mm × 5 mm subjected to laser heating and airflow-related convection.

### 3.2. Mechanical Analysis

To start with a simplified model, the following assumptions are made for this static case of a plate with a laser:

- The temperature field is axis symmetric to the center of the laser spot, with the distribution of a Gaussian function.
- The outer periphery of the plate was constrained with clamped boundary conditions.
- A linear condition is assumed with constant values of coefficient of thermal expansion and modulus.
- The in-plane analytical solution adopts plane stress, as in $\sigma_{z}=0$. This does not impose zero transverse strain.

With these assumptions, the mechanical analysis is simplified to an axisymmetric in-plane problem under the plane-stress assumption. The circular domain is a reference approximation for the response around a localized heating region. Its radial boundary condition does not reproduce the directional variation of the boundary distance in a square plate. This approximation is therefore not an exact solution for the complete square domain.

Equation (3) permits an elliptical temperature distribution. For the circular spots and loading conditions studied here, Equation (4) gives approximately equal fitted widths, $\sigma_{x}≃\sigma_{y}=\sigma$, so the mechanically relevant temperature input is nearly axisymmetric about the fitted center. The radial approximation in Equation (15) follows with $r^{2}={\left(x-\mu_{1}\right)}^{2}+{\left(y-\mu_{2}\right)}^{2}$, $a_{0}=B$, $a_{1}=A$, and $a_{2}=2\sigma^{2}$. It neglects the residual thermal asymmetry and is used as a first-order mechanical approximation.

This approximation is intended for the common circular laser spots considered here, with heating localized relative to the plate dimensions and the heated region sufficiently far from the edges. The centered validation cases have d/L=0.20 and 0.10, where *L* is the full plate side length. The comparisons with the three-dimensional finite element model in subsequent section support its use in the central region for the studied conditions. They do not establish a general spot-position tolerance or an error bound for strongly elliptical, markedly off-center, or near-edge heating.

The primary differential equation describes only the in-plane radial displacement. Out-of-plane deflection, curvature, and bending-induced strain are neglected. The learned thickness correction adjusts the in-plane strain magnitude for the sampled cases. It does not introduce a flexural degree of freedom or constitute a plate-bending model.

The equilibrium equations are as follows:

(9) $$\frac{\partial \sigma_{r}}{\partial r}+\frac{\sigma_{r}-\sigma_{\theta }}{r}=0$$

The constitutive equations are as follows:

(10) $$\sigma =C(\varepsilon -\varepsilon^{T})$$

Here, $\sigma ={\left[\sigma_{r},\sigma_{\theta },\sigma_{z}\right]}^{⊤}$ and $\varepsilon ={\left[\varepsilon_{r},\varepsilon_{\theta },\varepsilon_{z}\right]}^{⊤}$ are the normal-stress and total normal-strain vectors. For isotropic thermal expansion, $\varepsilon^{T}=\alpha \Delta T{\left[1,1,1\right]}^{⊤}$, where ΔT is the rise from the stress-free reference temperature. The normal-component block of the three-dimensional isotropic stiffness tensor is

(11) $$C=\frac{E}{\left(1+\nu )(1-2\nu \right)}\left[\begin{array}{ccc} 1-\nu & \nu & \nu \\ \nu & 1-\nu & \nu \\ \nu & \nu & 1-\nu \end{array}\right].$$

*E* is Young’s modulus and ν is Poisson’s ratio. Equation (11) is symmetric and uses the same isotropic constitutive law in cylindrical coordinates. The equivalent expanded form has diagonal entries $E(1-\nu^{2})/I$ and off-diagonal entries $E(\nu +\nu^{2})/I$, with $I={\left(1+\nu \right)}^{2}\left(1-2\nu \right)=1-3\nu^{2}-2\nu^{3}$.

The analytical reduction imposes plane stress, $\sigma_{z}=0$. Eliminating the transverse strain from Equations (10) and (11) gives the following in-plane constitutive relation:

$$\left[\begin{array}{c} \sigma_{r}\\ \sigma_{\theta } \end{array}\right]=\frac{E}{1-\nu^{2}}\left[\begin{array}{cc} 1 & \nu \\ \nu & 1 \end{array}\right]\left(\left[\begin{array}{c} \varepsilon_{r}\\ \varepsilon_{\theta } \end{array}\right]-\alpha \Delta T\left[\begin{array}{c} 1\\ 1 \end{array}\right]\right).$$

The transverse strain is generally nonzero and satisfies $\varepsilon_{z}=\alpha \Delta T-\nu \left(\sigma_{r}+\sigma_{\theta }\right)/E$. The strains in Equation (12) are total in-plane strains. The temperature rise in the constitutive relation is $\Delta T=T-T_{\mathrm{ref}}$, where $T_{\mathrm{ref}}$ is the stress-free reference temperature.

The kinematic equations are as follows:

(12) $$\varepsilon_{r}=\frac{\partial u_{r}}{\partial r}\;\mathrm{and}\;\varepsilon_{\theta }=\frac{u_{r}}{r}$$

The boundary conditions are as follows:

(13) $$u_{r}\left(r=l\right)=0$$

Here, l=L/2 is the radius of the circular reference domain and *L* is the full side length of the square plate.

Substituting the plane-stress constitutive relation and Equation (12) into the radial equilibrium equation, Equation (9), gives

(14) $$\frac{d^{2}u_{r}}{dr^{2}}+\frac{1}{r}\frac{du_{r}}{dr}-\frac{u_{r}}{r^{2}}-\alpha \left(1+\nu \right)\frac{d\Delta T(r)}{dr}=0.$$

Here, α is the physical linear coefficient of thermal expansion. The factor (1+ν) follows from the plane-stress reduction. The radial temperature approximation obtained from Equation (3) is

(15) $$T\left(r,\theta \right)=a_{0}+a_{1}e^{-\frac{r^{2}}{a_{2}}}$$

Then, the non-homogeneous ordinary differential equation (ODE) of Equation (14) is solved using the method of variation of parameters. The detailed solution procedure is provided in Appendix A. The displacement field are then expressed as

(16) $$u_{r}=D_{1}r+\frac{D_{2}}{r}-\frac{\alpha \left(1+\nu \right)a_{1}a_{2}}{2r}\exp\left(-\frac{r^{2}}{a_{2}}\right).$$

In Equation (16), $u_{r}$ is the radial displacement and *r* is the radial distance from the fitted heating center. The symbols α and ν denote the coefficient of thermal expansion and Poisson’s ratio. In Equation (15), $a_{0}$ is the temperature offset, $a_{1}$ is the temperature amplitude, and $a_{2}$ is the squared spatial scale. The constants $D_{1}$ and $D_{2}$ are determined by regularity at r=0 and the constrained radial boundary at r=l, as given in Equations (17) and (18). No independent parameter *k* occurs in Equation (16).

To avoid singularity of the displacement at r=0,

(17) $$D_{2}=\frac{\alpha \left(1+\nu \right)a_{1}a_{2}}{2}.$$

With a clamped boundary condition of Equation (13),

(18) $$D_{1}=\frac{\alpha \left(1+\nu \right)a_{1}a_{2}}{2l^{2}}\left[\exp\left(-\frac{l^{2}}{a_{2}}\right)-1\right].$$

Then, the strain field can be determined as

(19) $$\varepsilon_{r}=\frac{du_{r}}{dr}=D_{1}-\frac{D_{2}}{r^{2}}+\alpha \left(1+\nu \right)a_{1}\left(1+\frac{a_{2}}{2r^{2}}\right)\exp\left(-\frac{r^{2}}{a_{2}}\right).$$

(20) $$\varepsilon_{\theta }=\frac{u_{r}}{r}=D_{1}+\frac{D_{2}}{r^{2}}-\frac{\alpha \left(1+\nu \right)a_{1}a_{2}}{2r^{2}}\exp\left(-\frac{r^{2}}{a_{2}}\right).$$

The apparent singularities in Equations (19) and (20) are removable. At the center, $\varepsilon_{r}\left(0\right)=\varepsilon_{\theta }\left(0\right)=D_{1}+\alpha \left(1+\nu \right)a_{1}/2$. Stresses can be evaluated from the plane-stress constitutive relation above, using the full temperature rise ΔT, including its uniform offset.

### 3.3. Comparison with Numerical Simulations

To validate the proposed theoretical approach for predicting the strain field in a metal plate subjected to combined laser irradiation and airflow, the theoretical results were benchmarked against results obtained from a coupled thermo-mechanical finite element analysis. The cases used in the following validation comparisons were excluded from the training of the machine learning models. The comparisons therefore assess the analytical reconstruction and the learned thickness correction on unseen loading and geometric conditions.

The numerical reference is a three-dimensional thermo-mechanical finite element model. The strains reported for comparison are in-plane strains extracted at the plate surface, not strains averaged through the thickness or evaluated at the mid-plane. The analytical model reconstructs these surface responses approximately from the measured back-surface temperatures. It does not resolve the separate membrane and bending contributions to the numerical surface strain.

(1) Case 1: plate length *L* = 100 mm, thickness *z* = 2 mm, Ma = 0.6, laser power 2 kW, spot diameter *d* = 20 mm.

The material properties of mechanical behavior are included in the numerical model in addition to their thermal properties, with a modulus of 70 GPa, Poisson’s ratio of 0.33, and thermal expansion of 2.3 × 10^−5^/°C.

Temperature data at discrete measurement points on the back surface were collected from the numerical simulations. The Gaussian fitting parameters in Equation (15) for the temperature field are $a_{0}$ = 317.2, $a_{1}$ = 107.9, and $a_{2}$ = 678.6. Then, with these parameters, the displacement and strain field can be calculated using Equations (16)–(20).

**Figure 6:**
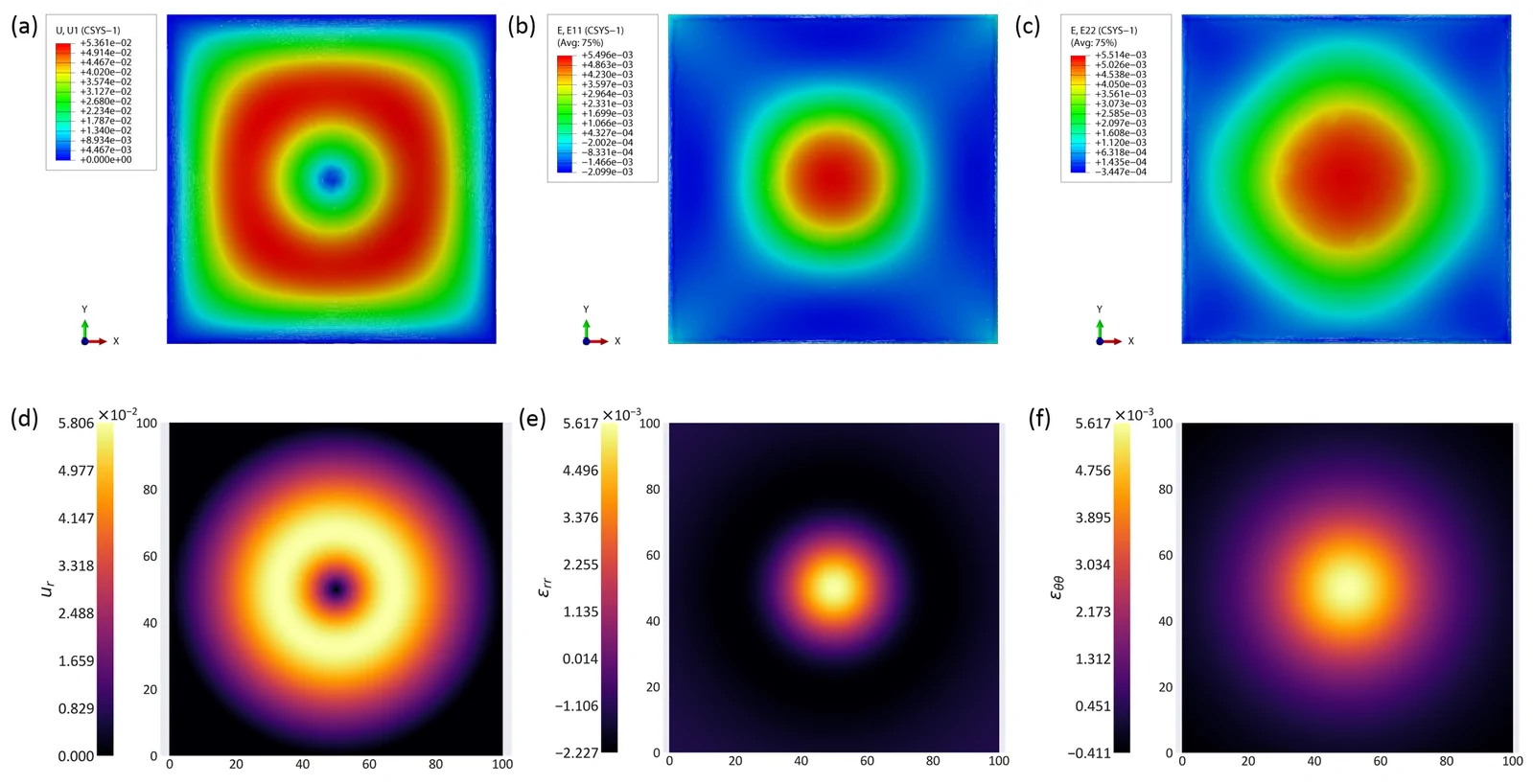
Comparison between theoretical and numerical models for displacement and strain fields in Case 1. (**a**–**c**) Numerical model. (**d**–**f**) Theoretical model.

The contour maps of temperature and in-plane strain predicted by the numerical model and the theoretical model are compared in Figure 6. According to the numerical results, the displacement and strain fields exhibit an approximately axisymmetric pattern over most of the plate. From the plate center to the boundary, the displacement increases gradually from zero and then decreases to zero at the boundary, with the maximum displacement occurring at an intermediate radius away from the center. In terms of in-plane strain, the strain decreases monotonically from the center toward the boundary. It shows tensile strain near the center and progressively transitions to compressive strain approaching the edge. Comparing the theoretical predictions with the numerical contours, both the peak displacement and the maximum in-plane strain show good agreement, with discrepancies within 10%.

The square and circular domains differ in the geometry of their constrained boundaries. For a square plate, the distance from the center to the boundary varies with direction and is greater toward a corner than toward a side midpoint. The circular model replaces these different distances by a single radial boundary. Consequently, differences near the edges and corners should be interpreted as effects of boundary geometry and non-axisymmetric constraint. A constrained corner is not a free boundary at which the stress must vanish, and corner stress relaxation is not a general consequence of the square geometry. The circular solution should be interpreted as a local reference approximation. This geometric explanation alone does not establish a quantitative error bound or validity range.

(2) Case 2: plate length *L* = 300 mm, thickness *z* = 5 mm, Ma = 0.2, laser power 3 kW, spot diameter *d* = 30 mm.

**Figure 7:**
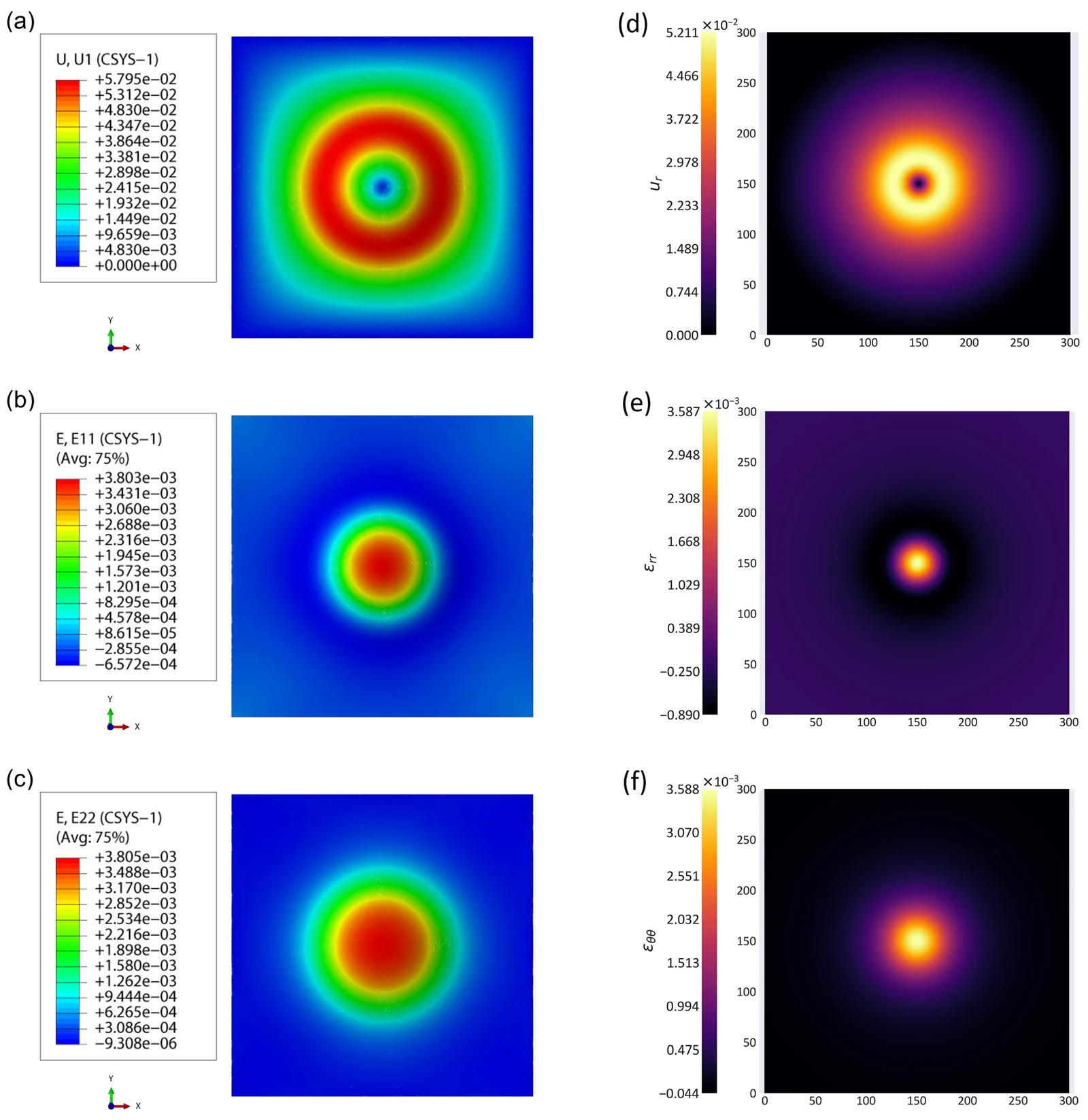
Comparison between the theoretical and numerical models for displacement and strain fields in Case 2. The left column shows numerical results (**a**–**c**), and the right column shows theoretical results (**d**–**f**). The rows show radial displacement, radial strain, and circumferential strain, respectively.

For Case 2, it can be observed from Figure 7 that, reducing the spot size relative to the plate dimensions yields displacement and strain fields that are closer to axisymmetric, indicating that the proposed theoretical model is more applicable to large-scale structures. Similarly, the theoretical model predicts the extrema of both displacement and strain with good accuracy, with errors within 10%.

**Figure 8:**
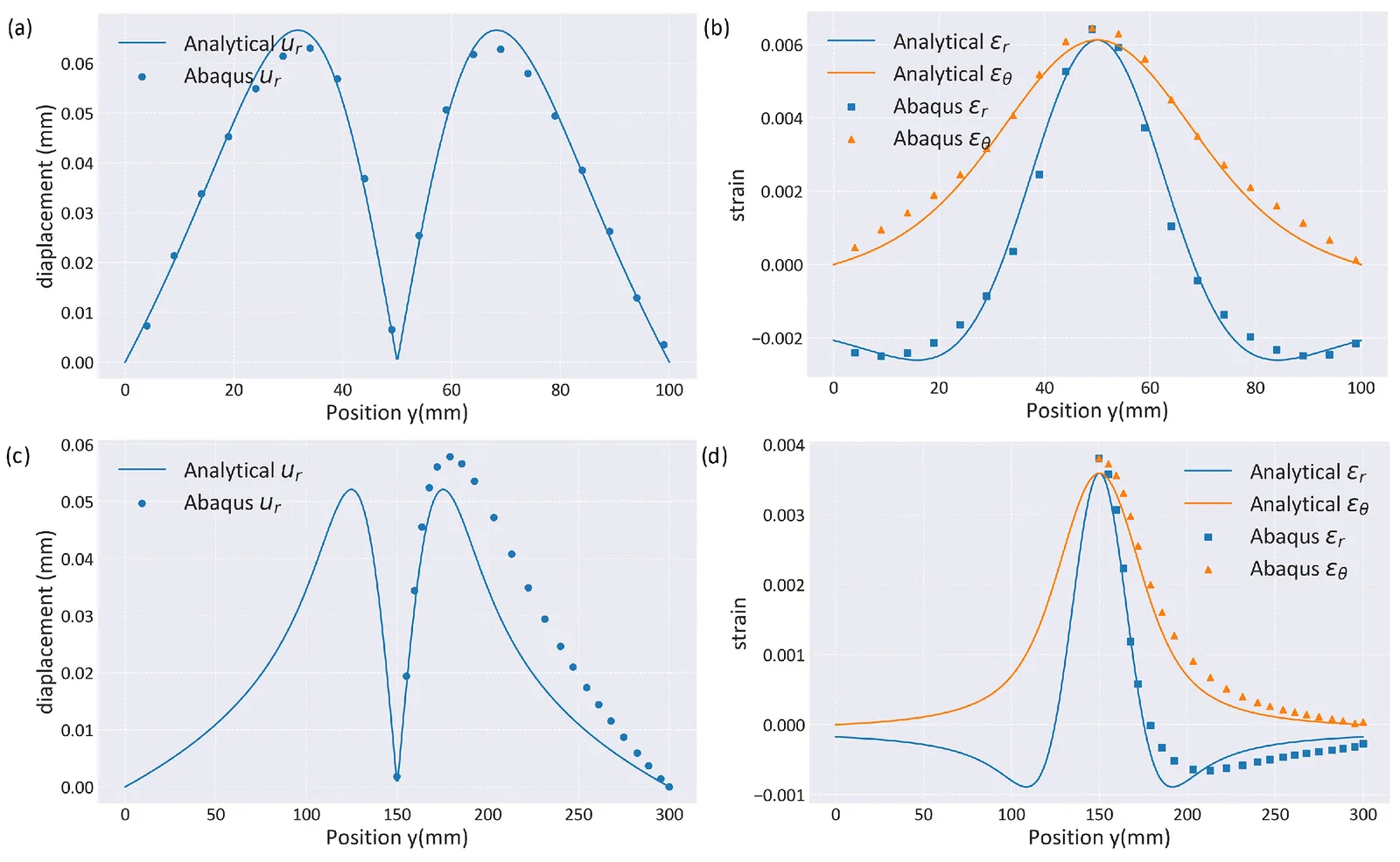
Comparison between theoretical and numerical model for displacement and strain curves at the central line. (**a**,**b**) Case 1. (**c**,**d**) Case 2.

To further assess the accuracy of the theoretical model, profiles along the midline section of the plate were extracted from the simulations, as shown in Figure 8. For the displacement field, the theoretical prediction captures the overall trend and spatial pattern. While in Case 1 good accuracy can be found, it underestimates the numerical results at regions away from the center in Case 2. This discrepancy primarily arises because the temperature on the laser-irradiated front surface remains higher than that on the back surface, whereas the present model does not account for the through-thickness temperature gradient. The discrepancy of the displacement field further causes underestimates of the strain at the central region. When the thickness is smaller, the difference is less significant, as suggested by the comparison between two cases in Figure 8. These comparisons illustrate the response captured by the approximation in the considered cases. They should not be interpreted as establishing uniform accuracy over the entire square plate.

### 3.4. Thickness Correction Factor by Data-Driven Analysis

As the results indicate, the current analytical formulation is derived from a two-dimensional axisymmetric thermoelastic model, in which the temperature field is treated as a radial distribution prescribed on a representative surface. While this framework captures the overall spatial trend of the response with high efficiency, the practical laser-heating problem is inherently three-dimensional, with a pronounced through-thickness temperature gradient that develops under surface irradiation. It leads to the fact that the near-surface expansion driving the in-plane strain is higher than that inferred from the back-surface temperature field. Consequently, using the back-surface temperature as the analytical input tends to underestimate the strain magnitude compared with the numerical predictions. This is evidenced by the fact that the discrepancy is lower with thinner plates.

To account quantitatively for this thickness-induced discrepancy, interpretable machine learning is used to identify a closed-form correction factor from the differences between the analytical and three-dimensional numerical responses. Symbolic regression searches for the mathematical structure and coefficients of the correction as functions of the geometric parameters. This provides an explicit, inspectable relation that augments the thermoelastic model and retains its computational efficiency.

A common factor is used for the radial and circumferential strains because the correction targets the central stress-concentration region. As shown in Figure 8, these two strain components are close to each other in this region in both the numerical results and the theoretical predictions. Their similar local response motivates correcting the shared underestimation of their magnitudes with the same factor. This local observation does not imply that the two strain components are identical throughout the plate.

The corrected strain field can be calculated as

(21) $$\bar{\varepsilon_{r}}=\varepsilon_{r}\cdot g(z,d,l)$$

(22) $$\bar{\varepsilon_{\theta }}=\varepsilon_{\theta }\cdot g(z,d,l)$$

**Figure 9:**
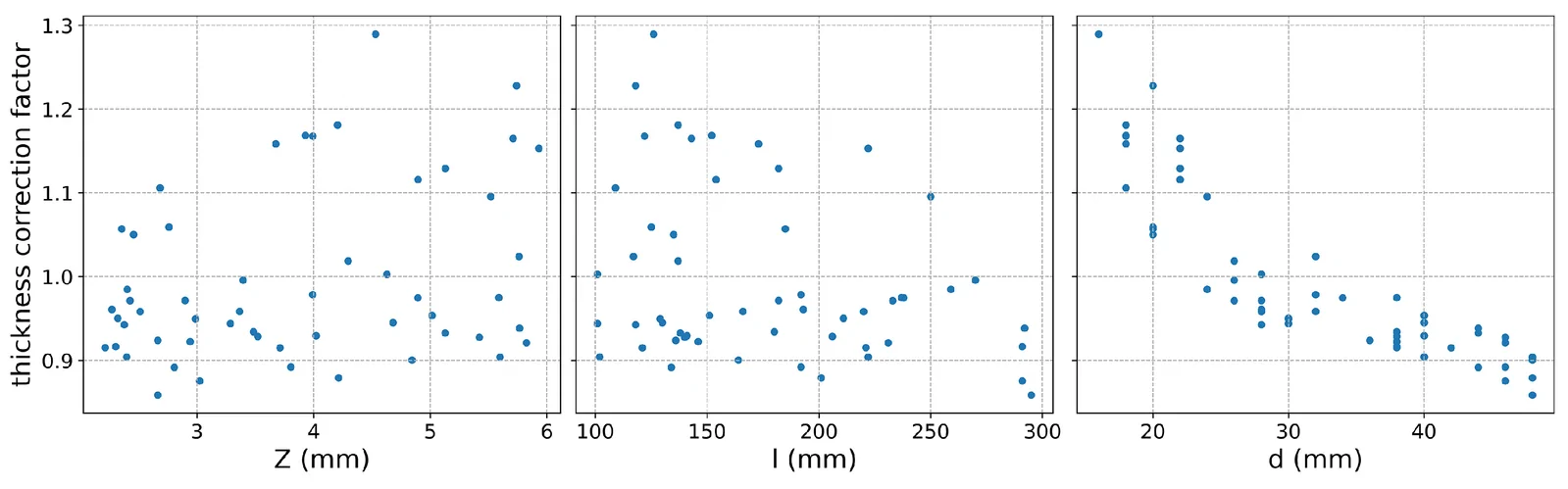
The relation between the thickness correction factor and the three geometric parameters of thickness, plate length, and spot diameter in the training dataset.

The functional form of this correction was then identified in a data-driven manner by fitting to the high-fidelity numerical results for square plates, thereby embedding thickness effects into the interpretable analytical surrogate. In order to do so, a python script was used to collect data from FEM simulations of plates with different sizes for thickness z∈[2,5] mm, full plate length L=2l∈[100,300] mm and spot diameter d∈[10,50] mm. In each case, in order to emphasize on the central part of the strain field where the maximum strain locates, data points were collected at the region where the ratio of numerical values to theoretical predictions are calculated. The total 100 data points are illustrated in Figure 9, with three plots representing the relationships between the ratio and the three geometric parameters. It can be seen that there is almost no dependence of the thickness correction factor on one single parameter.

Then, the symbolic regression model was utilized to capture the possible complex formulation of g(z,d,l). A similar model as presented in Figure 4. The training iterations are 200 and the maximum complexity of the PySR model is set to be 12. The square and square root are included in addition to the basic operators. According to the symbolic regression analysis, the geometric factor can be written as

(23) $$g\left(z,d,l\right)={\left(\frac{\sqrt[{4}]{z}}{-z+d}+0.941\right)}^{4}$$

**Figure 10:**
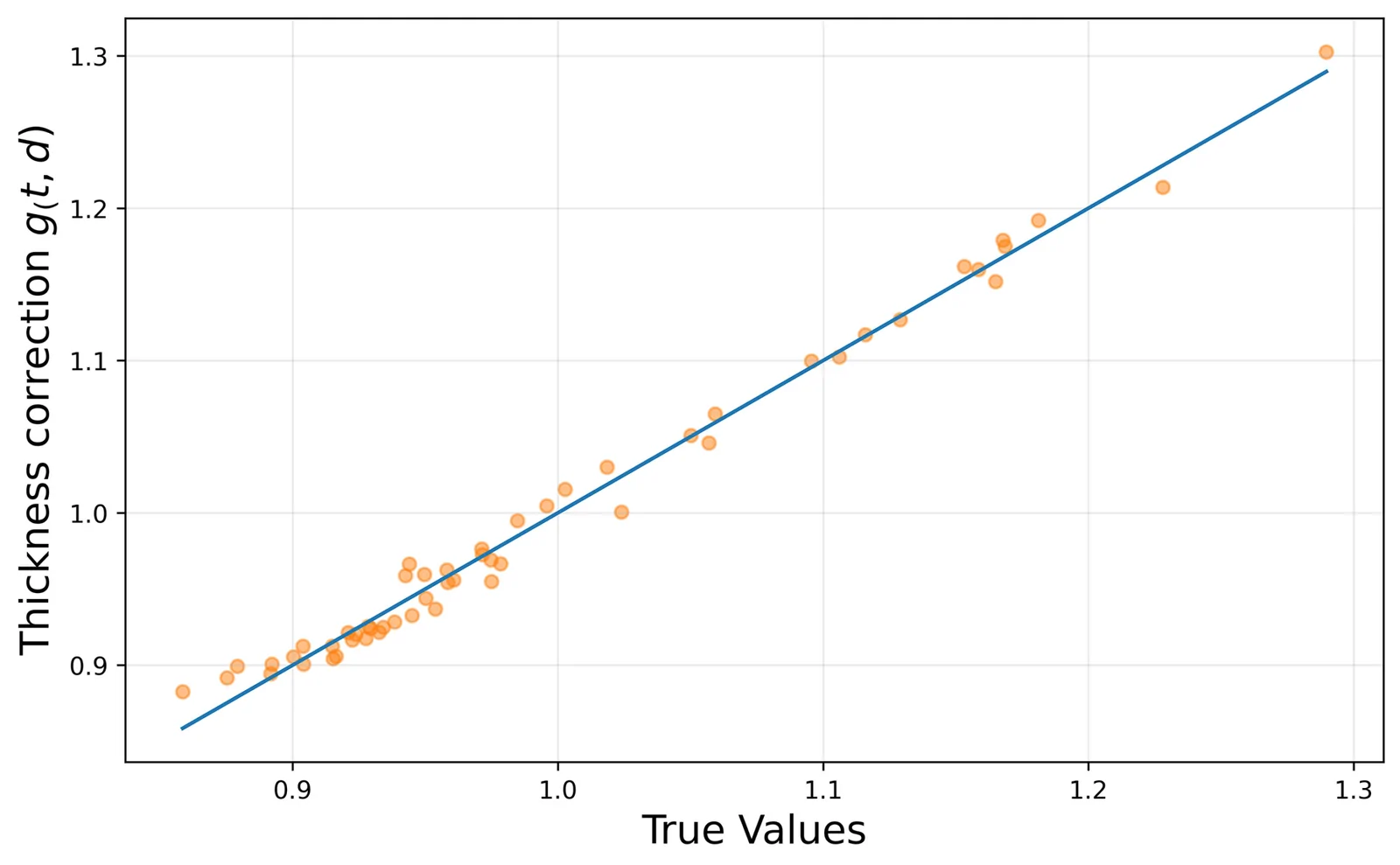
Regression performance of the proposed thickness-correction factor g(z,d,l).

Equation (23) is a quantitative correction identified by interpretable symbolic regression for the stated geometric ranges. The complete fourth-power expression is nonnegative wherever it is finite. The singularity at z=d lies outside the studied ranges of z=2–5 mm and d=10–50 mm. Its use is restricted to the stated parameter domain. The regression performance of the proposed g(z,d,l) is shown in Figure 10. The model is able to explicitly express the quantitative relationship because the R-square score is over 0.9.

The explicit dependence on thickness and spot diameter makes the learned correction interpretable in terms of the geometric scales associated with three-dimensional heat conduction. Its contribution is to convert discrepancies between the baseline theory and numerical responses into a compact quantitative relation. The absence of plate length from the selected expression pertains to the sampled conditions and the central region used for learning.

**Figure 11:**
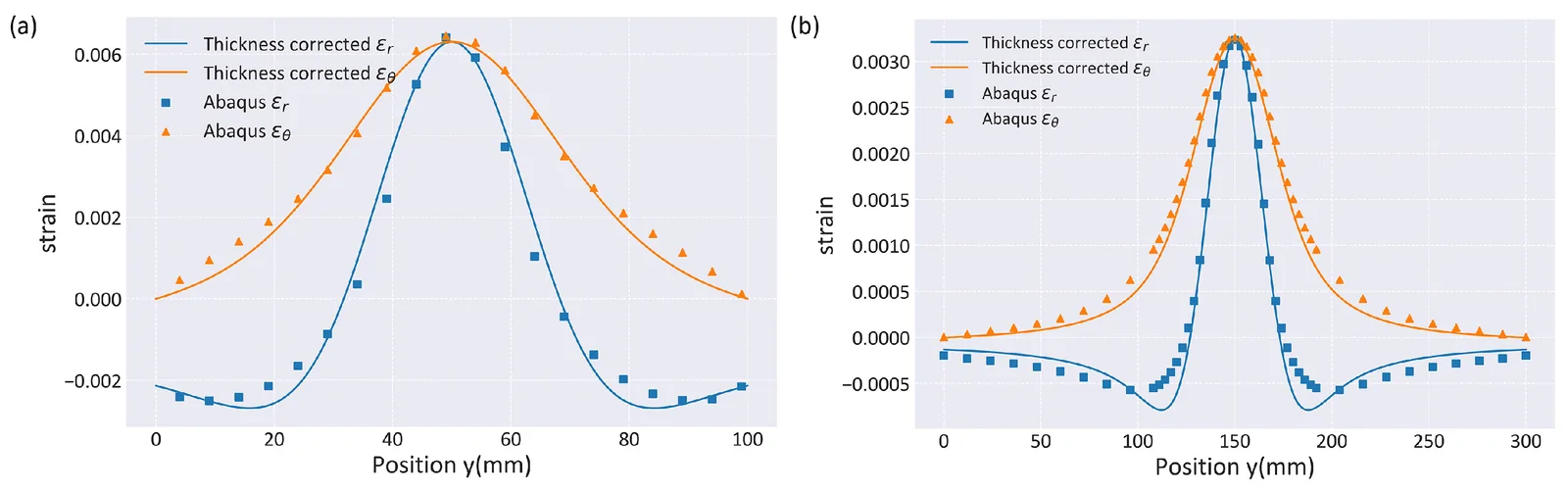
Displacement and strain curves of the theoretical model with thickness-correction factor. (**a**) Case 1. (**b**) Case 2.

After applying the thickness correction factors following Equations (21)–(23), the corrected results of the two cases in Figure 8 are presented in Figure 11. Compared to the previous model, with the use of the thickness-correction factor, the strain, especially the maximum strain at the central region of the plate, has been greatly improved.

After offline training, laser-parameter recovery and strain-field prediction from the limited temperature measurements can be completed in less than 1 s per case in the present implementation. This online evaluation time excludes finite element data generation and symbolic-regression training.

### 3.5. Preliminary Examples with Temperature-Dependent Material Properties

The primary closed-form solution assumes temperature-independent material properties. To retain an initial assessment of this limitation, two illustrative numerical examples are considered here. They explore temperature-dependent expansion and stiffness and are not presented as a generally validated nonlinear constitutive extension. The prescribed property laws are

(24) $$\alpha \left(T\right)=\alpha_{0}+m_{1}\left(T-T_{0}\right),$$

(25) $$E\left(T\right)=\frac{E_{0}}{1+m_{2}\left(T-T_{0}\right)+m_{3}{\left(T-T_{0}\right)}^{2}}.$$

Both examples use $T_{0}=300$ K. For the aluminum example, $\alpha_{0}=2.3\times 10^{-5}$ K^−1^, $E_{0}=70$ GPa, $m_{1}=6.0\times 10^{-9}$ K^−2^, $m_{2}=1.0\times 10^{-4}$ K^−1^, and $m_{3}=3.6\times 10^{-6}$ K^−2^. For the titanium example, $\alpha_{0}=8.6\times 10^{-6}$ K^−1^, $E_{0}=116$ GPa, $m_{1}=2.0\times 10^{-9}$ K^−2^, $m_{2}=1.0\times 10^{-3}$ K^−1^, and $m_{3}=3.0\times 10^{-7}$ K^−2^. These parameter sets define the illustrative calculations and are not asserted to be experimentally calibrated property data for specific alloy grades.

**Figure 12:**
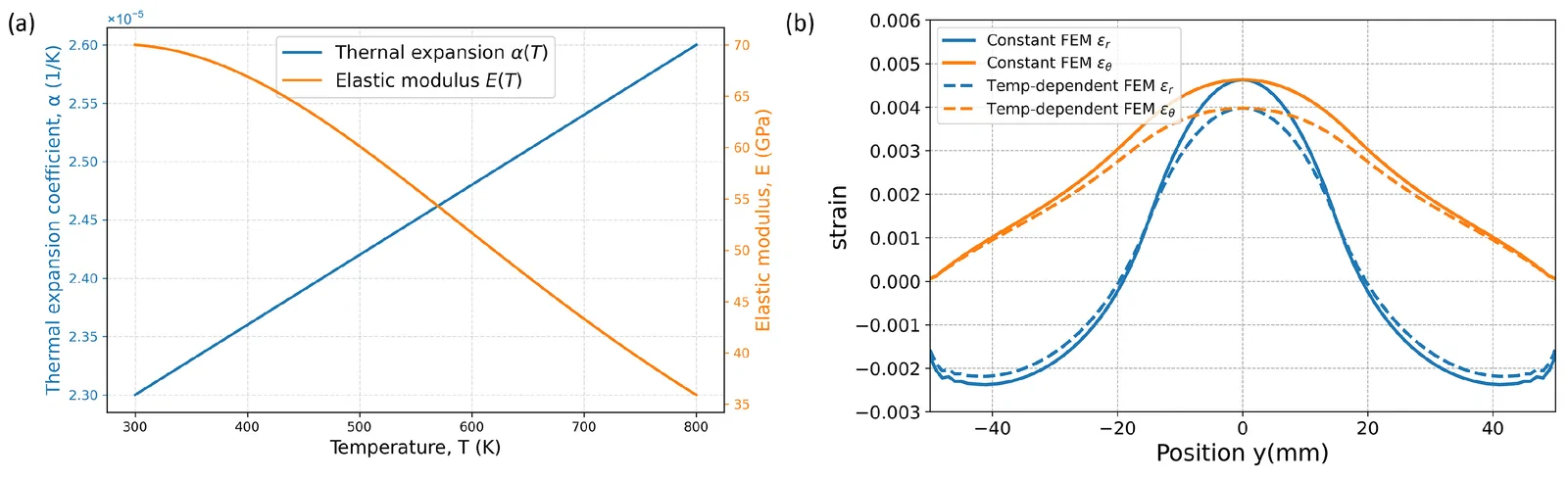
Preliminary aluminum example. (**a**) Prescribed temperature-dependent properties. (**b**) Constant-property and temperature-dependent finite element strain profiles.

**Figure 13:**
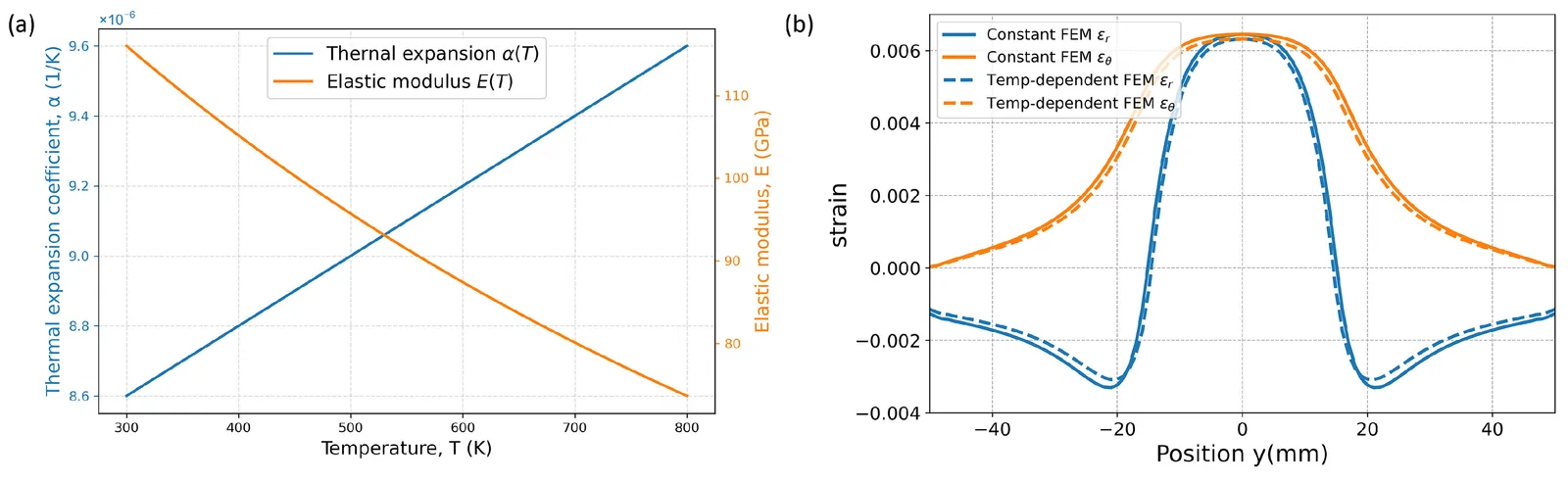
Preliminary titanium example. (**a**) Prescribed temperature-dependent properties. (**b**) Constant-property and temperature-dependent finite element strain profiles.

**Figure 14:**
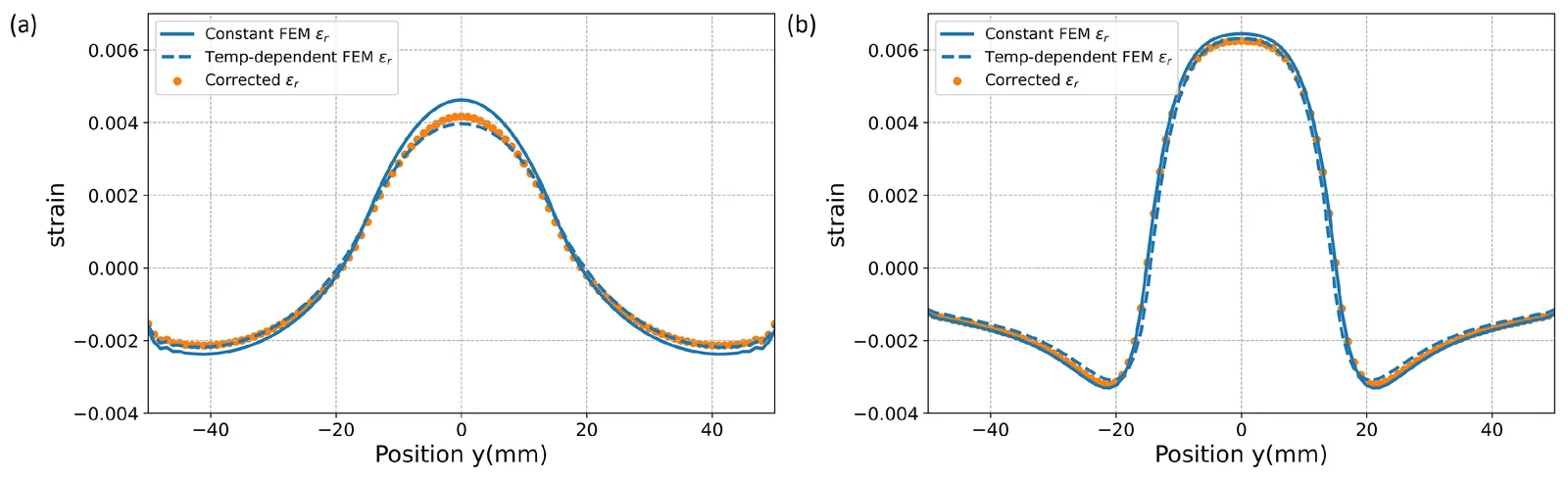
Illustrative case-specific scaling of the constant-property radial strain. (**a**) Aluminum with $c_{\mathrm{mat}}=0.90$. (**b**) Titanium with $c_{\mathrm{mat}}=0.97$. These comparisons are not independent validations of a general material correction.

Figure 12 and Figure 13 compare the constant-property and temperature-dependent finite element responses. The aluminum example shows a reduction of the peak in-plane strain from approximately $4.6\times 10^{-3}$ to $4.0\times 10^{-3}$, whereas the change is smaller in the titanium example. To illustrate a simple local adjustment, Figure 14 uses $\varepsilon_{r}^{\mathrm{ill}}=c_{\mathrm{mat}}\varepsilon_{r}^{\mathrm{const}}$, where $\varepsilon_{r}^{\mathrm{const}}$ is the constant-property numerical reference and $c_{\mathrm{mat}}=0.90$ for aluminum and 0.97 for titanium. These factors were chosen for the displayed cases. This illustration is distinct from the symbolic-regression thickness correction in Section 3.4 and does not demonstrate transfer to unseen material laws or loading conditions. A consistent general extension would require the temperature-dependent constitutive relations and separate validation.

## 4. Conclusions

This work is motivated by the need for accurate yet efficient predictive tools for thermal-mechanical responses of structural components subjected to combined laser irradiation and airflow, as encountered in aerospace and other extreme thermal environments. The following work has been presented in this paper.

- A theoretical model is proposed to predict the thermal–mechanical response under combined laser and airflow loading. Starting from array temperature measurements, the temperature field and the corresponding strain field of the plate can be reconstructed and evaluated.
- Interpretable symbolic regression establishes explicit relations between the fitted temperature-field parameters and the laser conditions. These relations can be rearranged to recover laser position, spot diameter, and incident power within the stated parameter range when the required airflow condition and normalization are known.
- A quantitative thickness correction is obtained through interpretable symbolic regression to incorporate discrepancies associated with three-dimensional heat conduction into the baseline thermoelastic model. The learned closed-form factor improves the radial and circumferential strain predictions in the central region of the unseen validation cases.
- Preliminary numerical examples illustrate the influence of temperature-dependent material properties and the potential for case-specific strain scaling. A general nonlinear constitutive extension has not yet been validated.

From an engineering perspective, the framework combines discrete temperature information, thermoelastic analysis, and interpretable machine learning to support structural response reconstruction and laser-parameter estimation. Numerical validation on unseen cases demonstrates the predictive performance under the modeled thermal boundary conditions. Experimental assessment, uncertainty analysis of recovered parameters, and matched hardware timing comparisons with full finite element simulation are needed to establish performance in a practical real-time monitoring system.

From a methodological standpoint, this study demonstrates that interpretable machine learning or XAI tools can serve as a mechanism for knowledge discovery. The presented workflow, starting from a transparent baseline model and augmenting it via explainable, explicit correction terms learned from data, provides a general and extensible paradigm. It may offer heuristic guidance for future theoretical developments and model construction in the complex scenarios involving coupled physics, nonlinear material behavior, and non-ideal boundary conditions.

## Data Availability

The original contributions presented in this study are included in the article. Further inquiries can be directed to the corresponding author.

## Appendix A. ODE Solution for the Axisymmetric Plane-Stress Problem

For compact notation, define $q=\alpha \left(1+\nu \right)a_{1}$. Substitution of the Gaussian temperature field into Equation (14) gives the forcing $f\left(r\right)=-2qr\exp\left(-r^{2}/a_{2}\right)/a_{2}$. The associated homogeneous equation has two linearly independent solutions:

(A1) $$u_{r1}=r,\;u_{r2}=\frac{1}{r}.$$

The general homogeneous solution is

(A2) $$U_{r}=c_{1}r+\frac{c_{2}}{r}.$$

For variation of parameters, replace the constants by $c_{1}\left(r\right)$ and $c_{2}\left(r\right)$. They satisfy

(A3) $$rc_{1}^{\prime }\left(r\right)+\frac{c_{2}^{\prime }\left(r\right)}{r}=0,$$

(A4) $$c_{1}^{\prime }\left(r\right)-\frac{c_{2}^{\prime }\left(r\right)}{r^{2}}=f\left(r\right)=-\frac{2qr}{a_{2}}\exp\left(-\frac{r^{2}}{a_{2}}\right).$$

Thus $c_{1}^{\prime }=f/2$ and $c_{2}^{\prime }=-r^{2}f/2$. Integration gives

(A5) $$c_{1}\left(r\right)=\frac{q}{2}\exp\left(-\frac{r^{2}}{a_{2}}\right)+D_{1},$$

(A6) $$c_{2}\left(r\right)=-\frac{q}{2}\left(a_{2}+r^{2}\right)\exp\left(-\frac{r^{2}}{a_{2}}\right)+D_{2}.$$

The terms proportional to $r\exp(-r^{2}/a_{2})$ cancel when these expressions are substituted into $u_{r}=c_{1}\left(r\right)r+c_{2}\left(r\right)/r$. Therefore,

(A7) $$u_{r}=D_{1}r+\frac{D_{2}}{r}-\frac{qa_{2}}{2r}\exp\left(-\frac{r^{2}}{a_{2}}\right).$$

Regularity at the center requires

(A8) $$D_{2}=\frac{qa_{2}}{2}.$$

The constrained radial boundary is

(A9) $$u_{r}\left(l\right)=0.$$

Consequently,

(A10) $$D_{1}=\frac{qa_{2}}{2l^{2}}\left[\exp\left(-\frac{l^{2}}{a_{2}}\right)-1\right].$$

The regular displacement solution can also be written as

(A11) $$u_{r}=D_{1}r+\frac{qa_{2}}{2r}\left[1-\exp\left(-\frac{r^{2}}{a_{2}}\right)\right].$$

Differentiating this expression and dividing it by *r* recover Equations (19) and (20). As r→0, $u_{r}/r$ and $du_{r}/dr$ both tend to $D_{1}+q/2$.
